# Conceptual foundations of acetylcarnitine supplementation in neuropsychiatric long COVID syndrome: a narrative review

**DOI:** 10.1007/s00406-023-01734-3

**Published:** 2024-01-03

**Authors:** Dario Lucas Helbing, Eva-Maria Dommaschk, Lena Vera Danyeli, Edgars Liepinsh, Alexander Refisch, Zümrüt Duygu Sen, Liga Zvejniece, Tonia Rocktäschel, Leonie Karoline Stabenow, Helgi B. Schiöth, Martin Walter, Maija Dambrova, Bianca Besteher

**Affiliations:** 1 Department of Psychiatry and Psychotherapy, Jena University Hospital, Friedrich Schiller University Jena, Philosophenweg 3, 07743 Jena, Germany; 2 Laboratory of Pharmaceutical Pharmacology, Latvian Institute of Organic Synthesis, Riga, Latvia; 3 Center for Intervention and Research on Adaptive and Maladaptive Brain Circuits, Underlying Mental Health (C-I-R-C), Jena, Magdeburg, Halle, Germany; 4 Department of Surgical Sciences, Functional Pharmacology and Neuroscience, Uppsala University, 751 24 Uppsala, Sweden; 5 German Center for Mental Health (DZPG), Site Halle, Jena, Magdeburg, Germany; 6 Center for Behavioral Brain Sciences, Magdeburg, Germany; 7 Department of Behavioral Neurology, Leibniz Institute for Neurobiology, Magdeburg, Germany; 8 Max Planck Institute for Biological Cybernetics, Tübingen, Germany; 9 Leibniz Institute on Aging, Fritz Lipmann Institute, Jena, Germany; 10 Institute of Molecular Cell Biology, Jena University Hospital, Friedrich Schiller University Jena, 07745 Jena, Germany; 11 Department of Anaesthesiology and Intensive Care Medicine, Jena University Hospital, Friedrich Schiller University Jena, Jena, Germany; 12 Department of Psychiatry and Psychotherapy, University Tübingen, Tübingen, Germany; 13 Faculty of Pharmacy, Riga Stradins University, Riga, Latvia

**Keywords:** Acetylcarnitine, Long COVID, Fatigue, Depression, Cognitive dysfunction

## Abstract

Post-acute sequelae of COVID-19 can present as multi-organ pathology, with neuropsychiatric symptoms being the most common symptom complex, characterizing long COVID as a syndrome with a significant disease burden for affected individuals. Several typical symptoms of long COVID, such as fatigue, depressive symptoms and cognitive impairment, are also key features of other psychiatric disorders such as myalgic encephalomyelitis/chronic fatigue syndrome (ME/CFS) and major depressive disorder (MDD). However, clinically successful treatment strategies are still lacking and are often inspired by treatment options for diseases with similar clinical presentations, such as ME/CFS. Acetylcarnitine, the shortest metabolite of a class of fatty acid metabolites called acylcarnitines and one of the most abundant blood metabolites in humans can be used as a dietary/nutritional supplement with proven clinical efficacy in the treatment of MDD, ME/CFS and other neuropsychiatric disorders. Basic research in recent decades has established acylcarnitines in general, and acetylcarnitine in particular, as important regulators and indicators of mitochondrial function and other physiological processes such as neuroinflammation and energy production pathways. In this review, we will compare the clinical basis of neuropsychiatric long COVID with other fatigue-associated diseases. We will also review common molecular disease mechanisms associated with altered acetylcarnitine metabolism and the potential of acetylcarnitine to interfere with these as a therapeutic agent. Finally, we will review the current evidence for acetylcarnitine as a supplement in the treatment of fatigue-associated diseases and propose future research strategies to investigate the potential of acetylcarnitine as a treatment option for long COVID.

## Introduction

Coronavirus disease 2019 (COVID-19) is associated with neuropsychiatric symptoms both during the acute SARS-CoV-2 infection as well as within weeks to months after recovery from the acute disease episode, termed post-acute sequelae of SARS-CoV-2 infection (PASC) [23]. PASC that persist for more than 4 weeks after the acute infection are termed “long COVID” [12, 26, 31], with varying incidences between 10 and 70% of infected COVID-19 patients developing long COVID symptoms, depending on the study population examined, e.g., non-hospitalized, hospitalized or vaccinated patients, etc. [31]. Long COVID is a multi-system disease affecting heart, lungs, gastrointestinal tract, kidneys, spleen and the liver, as reviewed in detail elsewhere [6, 26, 31]. However, neuropsychiatric symptoms developing on the basis of direct brain tissue pathology or underlying vascular dysfunction are considered to be a major and the most common feature of long COVID [4, 7, 31, 37, 65, 88, 113], with potentially devastating consequences for affected individuals. In particular, fatigue with post-exertional malaise, cognitive impairment characterized by deficits in executive functions, concentration and memory and depressed mood [88] are the main features of neuropsychiatric long COVID syndrome [31], with fatigue being the most common and debilitating [99].

It is interesting to note that other post-acute infection syndromes (PAIS) and some neuropsychiatric diseases such as myalgic encephalomyelitis/chronic fatigue syndrome (ME/CFS), depression (especially the atypical/immunometabolic subtype (IMD) [86]) and others also have fatigue as a leading clinical symptom, which may serve as a model for investigating the mechanisms of fatigue and drawing conclusions regarding therapeutic options in long COVID. Indeed, most treatment options for long COVID, which focus on modulating neuroinflammation, vascular defects and energy metabolism alterations due to mitochondrial dysfunction, have been inspired by treatment approaches for PAIS and ME/CFS [31].

Acetylcarnitine belongs to a class of fatty acid metabolites called acylcarnitines, which are produced by the conjugation of fatty acids with l-carnitine, a physiological process essential for the transport of fatty acids into mitochondria for β-oxidation and ATP production [3, 29]. Interestingly, evidence from metabolomic studies has shown associations between abnormal acylcarnitine and acetylcarnitine levels in several of the aforementioned fatigue-associated neuropsychiatric diseases, as reviewed elsewhere [29]. Short-chain acylcarnitines, i.e., acylcarnitines with two to five carbon atoms, are the most abundant acylcarnitines in human plasma, with acetylcarnitine being the major acylcarnitine [29]. Consequently, acetylcarnitine supplementation has been shown to improve fatigue, depressive symptoms and cognitive dysfunction for example, by modulating mitochondrial metabolism and neuroinflammation in the aforementioned diseases [29]. Furthermore, acetylcarnitine supplementation has recently been proposed for the treatment of neuropsychiatric long COVID symptoms [115].

The aims of this review are (1) to provide an overview of the clinical and biological overlap between neuropsychiatric long COVID and other fatigue-associated neuropsychiatric disorders, (2) to examine the shared molecular mechanisms of fatigue and depressive symptoms involving acetylcarnitine metabolism, and finally (3) to review the current evidence on acetylcarnitine supplementation studies as a treatment for fatigue-associated syndromes and pharmacological aspects of acetylcarnitine supplementation to provide a conceptual foundation for acetylcarnitine supplementation in neuropsychiatric long COVID syndrome.

## Main part

### Similarities in clinical presentation of long COVID and post-acute infection syndromes as well as major depressive disorder with atypical features

Already in 2020 the first long COVID patients approached medical professionals from different disciplines [68]. At that time, physicians were often reluctant to diagnose this multifaceted clinical syndrome as long COVID because routine diagnostics of blood samples or imaging in survivors of mild acute COVID-19 rarely yielded any pathological results [120]. However, the diagnosis and treatment of patients with long COVID to date has made it very clear that the majority of patients experience a multi-system syndrome characterized by fatigue with post-exertional malaise, cognitive deficits, depressed mood, stress-related head and joint pain, insomnia and vegetative dysfunction [99]. So far, there is an alarming number of long COVID patients who do not recover after several months and even a subgroup with worsening symptoms over time [36, 126]. However, long COVID is only one of several post-acute infection syndromes (PAIS) that have been reported, not only after previous coronavirus epidemics (e.g., SARS-CoV-1 and MERS-CoV) but also after other systemic viral, bacterial and parasitic infections, as reviewed in [23]. According to their review, the clinical characteristics of other PAIS appear almost indistinguishable from long COVID as described above, including exertion intolerance, fatigue, neurocognitive and sensory impairment, sleep disturbances, joint and muscle pain, and non-specific symptoms that are often present but of variable severity.

Many of these PAIS patients, as well as PASC patients, even meet the criteria for ME/CFS after months of incomplete recovery, as has been impressively demonstrated in large registry studies after infections with H1N1/09 influenza A virus and varicella zoster virus [78, 139]. The significant clinical overlap between ME/CFS and PASC has very recently been reviewed here [82], with the hypothesis that both entities may be caused by a chronic state of multisystemic disequilibrium, including endocrinological, immunological, and/or metabolic changes.

ME/CFS is a frequent syndrome affecting 0.9% of the population [71]. It is diagnosed according to the Canadian consensus criteria, at least 6 months after onset when the following symptoms are present: fatigue, post-exertional malaise with a recovery period of at least 24 h, sleep dysfunction, pain, at least two neurological/neurocognitive symptoms and at least two symptoms involving the autonomic, neuroendocrine or immune system [19]. The underlying pathophysiology is mostly unresolved, with immunological factors often, but not exclusively, occurring after an initial infection with a distinct or gradual onset. Other putative origins investigated in large, population-based datasets include chronic autoimmune syndromes such as inflammatory bowel disease and psoriasis [136, 137], and even after thermal injury, especially in extensive injuries and on sun-exposed body parts [138].

To understand the biological overlap and therefore possible therapeutic targets for PAIS such as PASC, it is also interesting to note similar clinical features in a typical major depressive disorder, (MDD) also known as immunometabolic depression [86]. This syndrome is characterized by clinical symptoms of sickness behavior such as leaden paralysis of the limbs, hypersomnia, and hyperphagia/weight gain, while mood reactivity is unimpaired, i.e., mood brightens in response to positive events (according to DSM-5 criteria for MDD with atypical features), while still suffering from lack of concentration and higher levels of exhaustibility as in melancholic MDD [99]. Van Hoof et al. even reviewed clinical observations of behavioral, affective and cognitive features already 20 years ago and hypothesized that atypical depression is a secondary symptom in chronic fatigue syndrome [140].

Considering the clinical overlap of these multisystemic clinical syndromes, we will now proceed to summarize aspects of common biomarkers and pathological mechanisms underlying these shared symptoms.

### Shared pathomechanisms of neuropsychiatric syndromes with fatigue as a leading symptom (PASC, PAIS, ME/CFS, IMD)

#### Alterations of brain structure and connectivity

PASC is currently being investigated by neuroimaging experts all over the world. To date, structural imaging studies have reported inconsistent results, probably depending on the age of the participants, the severity of acute COVID-19 and the symptom burden at the time of scanning. The most consistent results so far are grey matter alterations in the basal ganglia, areas of the limbic system such as the anterior cingulate cortex, hippocampus, insula, etc., and prefrontal areas like the orbitofrontal cortex, sometimes even more pronounced in association with self-reported fatigue or neurocognitive impairment [12, 35, 49]. A recent longitudinal imaging study showed a reduction of thalamus and basal ganglia volumes as well as aberrant diffusion markers in post-COVID patients, which correlated with fatigue scores [50]. Regarding functional connectivity, several exploratory studies have confirmed a pattern of increased and/or decreased connectivity in frontotemporal networks associated with subjective cognitive impairment and olfactory dysfunction [98, 148, 152]. As demonstrated by [^18^F]FDG-PET/CT ([^18^F]Fluorodeoxyglucose positron emission tomography/computed tomography), patients suffering from long COVID also showed brain glucose hypometabolism in the right parahippocampal gyrus and thalamus, while specific hypometabolic area(s) characterized patients with persistent anosmia/ageusia, fatigue, and vascular uptake [119]. The brainstem, which has been implicated to be affected in PASC, explaining the broad spectrum of symptoms, particularly pain and autonomic symptoms [156], has been found to show glucose hypometabolism in patients (among other regions) [47, 53, 89]. Although technically challenging, this should be further investigated, especially in light of the neuroimaging findings in ME/CFS, described in the following paragraphs.

While there is no evidence of common structural or functional brain alterations in PAIS, probably due to its heterogeneous origin and lack of conceptualization, ME/CFS has been extensively studied with MRI (magnetic resonance imaging), as recently reviewed by Nelson et al. [94]. Although the findings are not always consistent, changes in the brainstem have been very common, especially structural changes, mainly a decrease in grey or white matter volume in patients, often pronounced in relation to the symptom burden of fatigue, pain and autonomic symptoms. One diffusion-tensor imaging study even showed decreased diffusivity in the brainstem and basal ganglia area in ME/CFS patients [129]. In addition, altered functional connectivity has also been reported in the brainstem, basal ganglia and related areas such as the limbic system [123]. Neuroinflammation, vascular dysfunction, and cellular dysfunction regarding astrocytes and neurons have been discussed as underlying mechanisms contributing to these structural and functional alterations [94]. Furthermore, ME/CFS patients showed glucose hypometabolism in the brainstem as well as in the right mediofrontal cortex, similar to findings in long COVID patients [130].

#### Neuroinflammation

Neuroinflammation is increasingly recognized as a key factor interacting with neurobiological correlates of the neuropsychiatric long COVID syndrome, such as brain serotonin depletion [111], hypothalamic–pituitary–adrenal (HPA) axis dysregulation [57], and impaired hippocampal neurogenesis and plasticity [13, 61].

The central nervous system (CNS) relies on a complex and intricate network of molecular and cellular interactions to maintain proper function and homeostasis. The initial SARS-CoV-2 viral infection triggers an immune response that may persist in some individuals even after the virus has been cleared, leading to ongoing hyper-inflammation. There is increasing evidence that neuroinflammation is an important component of ME/CFS [34] and probably also of long COVID [127]. Commonly suggested biomedical hypotheses to explain PAIS (such as in long COVID/PASC, ME/CFS) are chronic stimulation of the immune system by a persistent infection or persistent unviable pathogen structures; immune activation with targeting self-antigens, either due to infection-triggered impairment of regulatory T-cell function, molecular mimicry, dysregulation of the microbiota–gut–brain axis, as well as permanent organ damage [104]. The immune response to SARS-CoV-2 in the respiratory tract has been shown to induce neuroinflammation, which subsequently leads to an acute inflammatory response and immune cell trafficking in the brain, and induces reactive states of resident microglia and other immune cells in the brain and brain borders [44, 51, 154]. These processes may exist in combination and at different intensities in different patient subgroups. However, neuroinflammation and the consequent dysregulation of neural homeostasis and plasticity is likely to be a more common mechanistic principle, occurring even after mild COVID-19 disease [141]. Of note, neuroinflammation has furthermore been implicated in the pathophysiology of depression [87], whereas increased inflammatory markers, such as C-reactive protein (CRP) or interleukin-2 (IL-2), have been specifically described to be more associated with the atypical subtype of depression [69].

Perturbations in mitochondrial dynamics can affect many cellular and molecular pathways, particularly during inflammation [15] (for reviews, see [33, 116]). Inflammation stimulates the production of reactive oxygen species (ROS), which can further damage mitochondria and impair their function [103]. In addition, inflammation can alter the expression of genes involved in mitochondrial function, leading to changes in energy metabolism. Associations between mitochondrial genetic variation, activation of the immune system, and its effects on neurogenesis and neurotransmission have led some authors to suggest that mitochondrial dysfunction initiates a chain of molecular events leading to key symptoms of neuropsychiatric long COVID, such as depressive mood and cognitive impairment [39, 100, 135].

Of note, there are at least five other mechanisms besides secondary neuroinflammation by which COVID can affect the CNS (direct viral invasion of neural tissue, autoimmunity, reactivation of herpesviruses, neurovascular dysfunction, the sequelae of hypoxic, and other metabolic disturbances), which may occur exclusively or in combination in some individuals with varying frequency and timing (for review, see [88]).

Preclinical studies suggest that acetylcarnitine attenuates oxidative stress and neuroinflammation [2, 112]. Mechanisms that could potentially link acetylcarnitine to inflammatory pathways and synaptic activity include changes in monoaminergic and glutamatergic transmission. Such changes are commonly observed in neuropsychiatric disorders (for a review of these changes, see [153]). In brief, indoleamine 2,3-dioxygenase (IDO) activity is increased during inflammation, leading to increased conversion of tryptophan to kynurenine, reducing substrate availability for serotonin synthesis. Kynurenine is predominantly metabolized to the *N*-methyl-d-aspartate (NMDA) receptor agonist quinolinic acid (QUIN) in the inflammatory state, contributing to the accumulation of glutamate in the extracellular space, leading to changes in network connectivity associated with depression, fatigue and cognitive impairment. Acetylcarnitine, on the other hand, affects the epigenetic regulation of metabotropic glutamate type 2 receptors (mGluR2), negatively modulating neurotransmitter release and producing rapid and long-lasting antidepressant effects (demonstrated by forced swim tests, sucrose preference tests) in Flinders Sensitive Line rats and in mice exposed to chronic unpredictable stress, which model genetic and environmentally induced depression, respectively [92]. Using nuclear magnetic resonance (NMR) spectroscopy, Smeland and colleagues also demonstrated increased levels of norepinephrine and serotonin in the cortex of healthy mice, after 25 days of treatment with acetylcarnitine at a daily dose of approximately 0.5 g/kg body weight [117]. In addition, a non-enzymatic product of the prostaglandin D2 synthase gene (*PGD2S*), whose expression is upregulated by acetylcarnitine treatment with 100 mg/kg body weight [134], has been shown to have anti-inflammatory properties that may help protect the brain from ischemia–reperfusion injury [72].

Taken together, these findings suggest that acetylcarnitine supplementation may attenuate the inflammatory damage that contributes to neuropsychiatric long COVID syndrome.

#### Systemic metabolic alterations and their relationship to neuropsychiatric symptomatology

**Fig. 1:**
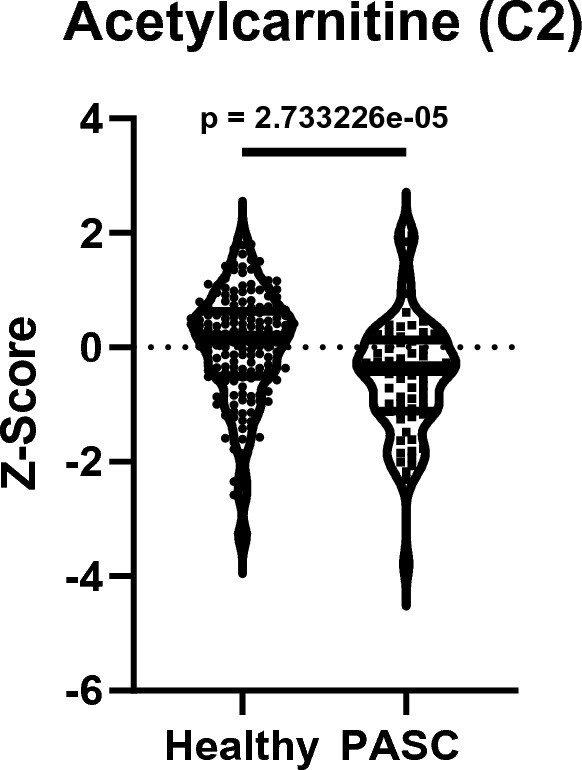
Metabolomic data from Su et al. [124] were retrieved from the provided Supplementary Data Set S2 (sheet “S2.2 Metabolomics”), as well as clinical data on PASC (post-acute sequelae of SARS-CoV-2 infection) symptoms (Table S1) from the INCOV cohort. Subsequently, *z*-score transformed metabolite concentrations for acetylcarnitine (C2) were extracted for healthy controls (*n* = 178) and all INCOV patients with ≥ 1 PASC symptom (*n* = 68), representing the PASC cohort. Shown are *z*-score transformed metabolite concentrations at T3, a time point 2–3 months after initial infection. *p* value represents the exact *p* value from a two-tailed Mann–Whitney test as the *z*-score transformed data were not normally distributed, calculated using GraphPad Prism 8.4.3. Violin plots show the median (bold black line) and quartiles (faint black lines)

PAIS, including long COVID, as well as CFS and IMD, share the characteristic of systemic metabolic disturbances, which may be a sign of underlying multi-organ inflammation and exhaustion [31], and consequently may contribute to neuropsychiatric symptomatology, as many metabolites can cross the blood–brain barrier (BBB) [30, 58] (e.g., short chain fatty acids (SCFAs) derived from the gut microbiota such as acetate [40], amino acids such as tryptophan [52] and also carnitine/acetylcarnitine can cross the BBB via transporters [59]). A large number of studies have investigated peripheral metabolomic changes in the aforementioned conditions, with considerable overlap between PAIS, CFS, and immunometabolic depression: As reviewed by Komaroff et al., the systemic metabolomic changes in ME/CFS patients are characterized by (1) an impairment in the production of ATP from all energy substrates, (2) a general “hypometabolic state” and (3) an imbalance between pro- and antioxidant metabolites favouring pro-oxidant processes [43, 62]. This metabolic dysregulation is defined by systemically reduced levels of metabolites such as amino acids and fatty acids, leading to reduced TCA cycle activity and oxidative phosphorylation, which may ultimately be a major contributor to neuropsychiatric symptoms associated with reduced energy, such as fatigue and other cognitive impairments [43, 62]. Interestingly, such signs of mitochondrial dysfunction have also been described in MDD and long COVID: de Boer et al. found that during exercise, patients with long COVID showed decreased rates of fatty acid oxidation [32], consistent with other reports [48] that describe a general metabolic profile of impaired mitochondrial fatty acid and lipid catabolism in general, as well as a decrease in several amino acids that can fuel mitochondrial TCA cycle activity. These findings have also been described for MDD, e.g., in a large meta-analysis by Bot and Milaneschi et al., which found an association between depression risk and different lipid classes [14] and in several other studies, as reviewed in detail by Guerreiro Costa et al. [74]. In addition to these general metabolic changes linking impaired energy production to fatigue as a common symptom between long COVID, ME/CFS and MDD, common differences in specific metabolites may also be of importance. Tryptophan (Trp) metabolism, and consequently Trp-derived metabolite pathways such as the kynurenine and serotonin pathways, have long been known to be central to the pathophysiology of MDD—lower blood concentrations of Trp have been described in several studies of MDD patients, and alterations in tryptophan and kynurenine levels have also been associated with symptomatology and treatment [24, 74]. In addition, the ratio of neuroprotective to neurotoxic kynurenine metabolites differs between healthy controls and ME/CFS patients [46], accompanied and probably often preceded by a general decrease in tryptophan [25, 74]. The direct relationship between tryptophan levels and depressive symptoms and fatigue has been demonstrated by Trp depletion experiments, as reviewed elsewhere [76], and is also of interest in the context of long-term COVID, as recent studies have shown a significant decrease in plasma Trp levels in PASC patients [48], and alterations in kynurenine pathway metabolites have been associated with cognitive impairment in long COVID patients [27], as well as with anxiety and depressive symptoms in COVID-19 survivors 6 months post-infection [66]. In addition to the aforementioned remodelling of the metabolome in fatigue-associated diseases, including long COVID, acylcarnitines have emerged as important modulators of CNS function [29]: In both mice and humans, different classes of acylcarnitines have been found to be altered in either mouse models of depression or humans with MDD, with associations to symptom severity and also treatment response, as reviewed by Liu et al. [73]. In particular, short-chain acylcarnitines, such as acetylcarnitine, which are thought to rather support neuronal function [29], were decreased in the plasma of MDD patients, and more harmful medium- and long-chain acylcarnitines were increased in several studies [73]. Interestingly, similar patterns have also been observed in ME/CFS [91] and long Covid patients [48]. Guntur et al. found that in a small cohort of PASC patients, increases in medium- and long-chain acylcarnitines were among the most pronounced changes in a plasma metabolomic experiment between healthy controls and patients with PASC [48]. However, short-chain acylcarnitines were not altered in this study, which may be explained by the relatively small patient cohort (29 PASC patients, 30 healthy controls) [48]. In contrast to this result, another recent study by Kovarik et al. found a significant decrease in C3-carnitine in patients with long COVID, although the cohort sizes in this study were also relatively small (13 healthy controls and 13 patients with long COVID) [63]. Therefore, we obtained the data from the—to our knowledge—largest plasma metabolomics study conducted to date in long COVID patients by Su et al. [124] (Table S2 from Su et al., sheet “S2.2 Metabolomics”, age-, sex- and BMI-adjusted z-score-transformed plasma metabolite concentrations) and re-analysed the plasma metabolomics data for acetylcarnitine to investigate differences in acetylcarnitine levels between healthy controls and patients with PASC (≥ 1 PASC symptom according to the clinical information available from Table S1, sheet “S1.3 PASC data” of Su et al.). Indeed, we observed a decrease in plasma acetylcarnitine levels in PASC patients (Fig. 1), which is consistent with previously reported decreases in acetylcarnitine in other fatigue-associated conditions and supports our hypothesis that acetylcarnitine supplementation may be beneficial in the treatment of long COVID-associated neuropsychiatric symptoms. Taken together, metabolomic studies of long COVID, ME/CFS, and MDD show considerable similarities in the overall metabolic profiles associated with a disruption of energy production and an imbalance of potentially neuroprotective and detrimental metabolites, i.e., a deficiency of neuroprotective metabolites such as acetylcarnitine.

#### Mitochondrial dysfunction in post-infectious fatigue

The SARS-CoV-2 virus primarily damages lung cells, thus affecting the respiratory system and causing acute respiratory distress, resulting in low blood and tissue oxygen levels. The pathways associated with hypoxia and dysregulated immune system have been identified as strong drivers of neurological complications of post-acute sequelae of SARS-CoV-2 infection (evidence from the post-mortem brain studies and observational studies) [97]. Global hypoxia affects the electron transport chain, fatty acid beta-oxidation and other metabolic pathways essential for mitochondrial energy production. Reduced ATP production and impaired mitochondrial function were observed not only in the lungs, but also in the heart and other tissues. In addition, COVID-19’s effects on the cardiovascular system reduce the availability of oxygen and nutrients throughout the body, depleting energy stores and causing energy deficency. Inefficient metabolism has been shown to lead to the accumulation of metabolic intermediates that can interfere with mitochondrial function and impair the ability to produce ATP. Several metabolic intermediates, such as long-chain acylcarnitines can cause oxidative stress at high levels and high levels of ROS can then cause damage to cells and tissues, leading to a further decline in performance. Overall, the biochemical mechanisms of COVID-19-induced tissue damage are complex and involve a range of metabolism-disturbing processes, including inflammation, hypoxia, energy deficiency, and accumulation of metabolic intermediates [45]. Accordingly, tissue damage of varying severity results in long recovery times after the acute phase of COVID-19 infection.

COVID-19 has now been classified as an endemic disease, but the consequences of COVID-19 infection manifest as long-term multiorgan dysfunction. Most attention is being paid to critical perturbations of the cardiovascular system, where the underlying mechanisms of post-COVID-19 cardiovascular dysfunction are associated with cardiac mitochondrial dysfunction [21]. Another syndrome is post-COVID-19 sarcopenia, characterized by loss of muscle mass and function [102], leading to exercise intolerance [8, 32]. The hyper-inflammatory response caused by SARS-CoV-2 induces the process of immunosenescence, increases endothelial cell damage, and leads to myofibrillar degradation and muscle wasting (sarcopenia) due to mitochondrial dysfunction and autophagy [102]. Persistent viral infection and inflammation may cause sleep disturbances observed 3 months after COVID-19, which may also be associated with mitochondrial dysfunction [128]. Several studies suggest that inflammation may contribute to depression by affecting the function of neuronal mitochondria in the brain [20]. Improving mitochondrial function has been shown to accelerate the resolution of post-COVID-19 symptoms [42, 125]. Overall, mitochondrial dysfunction may play a role in post-COVID-19 syndrome, and more research is needed to fully understand the underlying mechanisms and develop effective treatments, which is why we will elaborate further on acetylcarnitine metabolism in vivo and as a supplement.

### Acetylcarnitine metabolism in the context of post-infectious and/or inflammation-associated neuropsychiatric diseases

#### Biosynthesis and regulation of acetylcarnitine

**Fig. 2:**
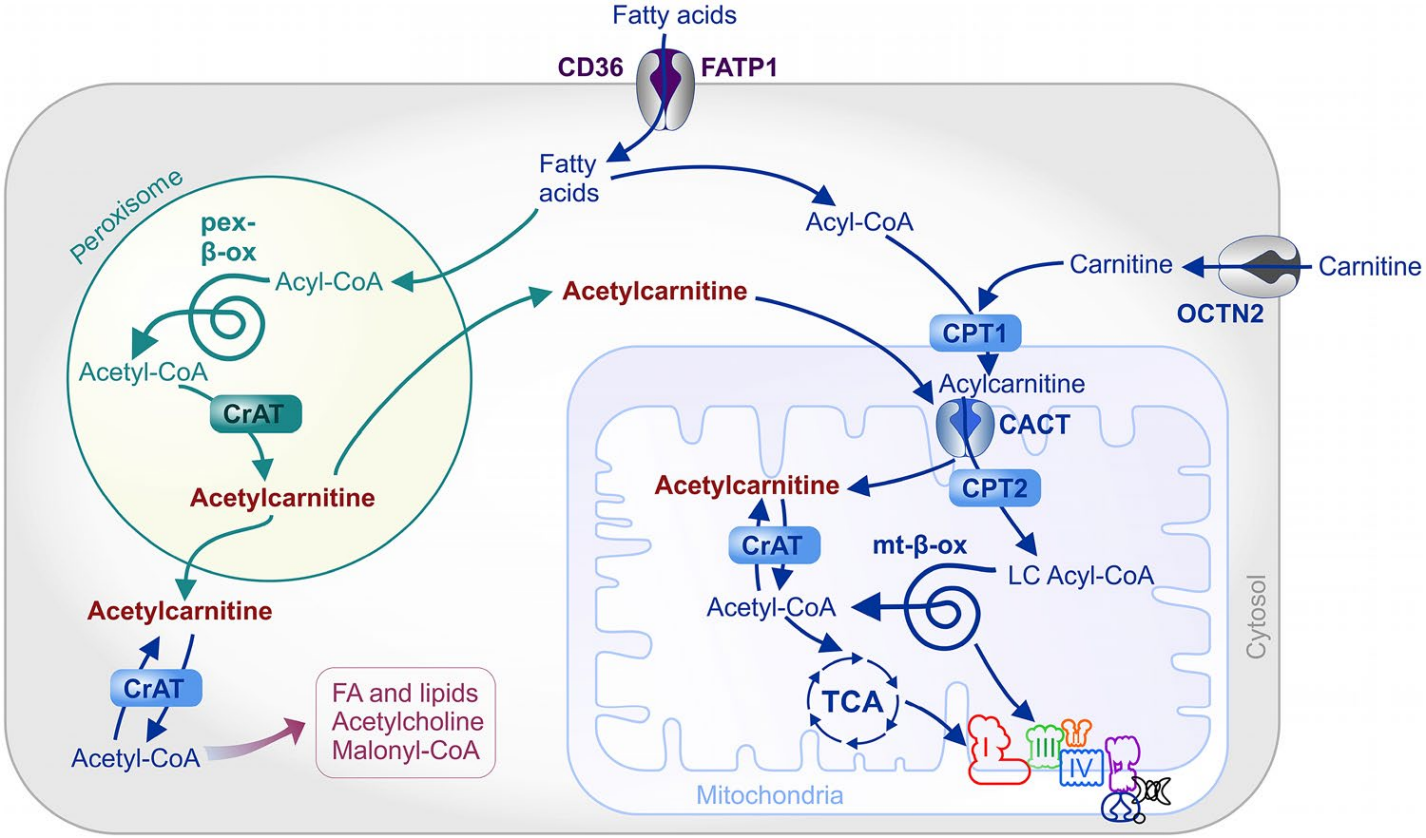
Synthesis and metabolism of acetylcarnitine. This figure is reproduced in part in a modified form under a CC BY-NC Attribution 4.0 International license from Dambrova et al. [29]

In the cell, acetylcarnitine is produced by conjugation of the acetyl group of acetyl-CoA with l-carnitine. The reaction is catalyzed by the carnitine acetyltransferase (CrAT, EC:2.3.1.7), which is present in many cell compartments, including the cytosol, the matrix or mitochondria and peroxisomes, as well as the endoplasmic reticulum and nucleus [5, 83, 106]. Carnitine for this reaction can be biosynthesized or obtained from dietary sources and further transported via the organic cation novel type 2 transporter (OCTN2) [75, 107, 122]. CrAT belongs to the family of carnitine acyltransferases, which ensures the transfer of different chain acyl groups to carnitine (Fig. 2). All acyltransferases carry out the bidirectional transfer of acyl groups between CoA and carnitine. CrAT is mainly capable of synthesizing short-chain acylcarnitines with up to five carbons in the acyl group. A less efficient reaction is possible for longer acyl groups with fatty acid chains of up to ten carbon atoms in length [144]. Peroxisomes metabolize a variety of fatty acids, acting as a chain-shortening β-oxidation system that produces large amounts of acetyl-CoA. As CrAT is present in the peroxisomes, peroxisomal CrAT converts a substantial amount of acetyl-CoA to acetylcarnitine [149]. In the cytosol, the reverse activity of CrAT could generate acetyl-CoA that is provided for a variety of biochemical reactions, such as the biosynthesis of malonyl-CoA, acetylcholine, and fatty acids [5].

In functional mitochondria, the supply of energy substrates is coupled with oxidative phosphorylation, which ensures a complete metabolic pathway for the generation of ATP. Thus, it can be estimated that only a small part of acetylcarnitine leaves the mitochondria and most of the available acetylcarnitine pool is generated in peroxisomes as a result of fatty acid breakdown. Unlike mitochondria, peroxisomes cannot further metabolize acetylcarnitine and therefore peroxisomes are a major source of acyl groups in the form of acetate and acetylcarnitine. In the bloodstream, acetylcarnitine concentrations are different in fed and fasted states, and accordingly acetylcarnitine availability is related to fatty acid load and metabolic rate, which is much higher in the fasted state. Furthermore, given that glucose and lactate metabolism in mitochondria is fairly complete, these energy substrates do not contribute to the available acetylcarnitine pool.

CrAT can perform reactions in both directions, therefore its activity depends on the acetyl-CoA/free CoA ratio and the availability of carnitine. For example, in mitochondria, when the rate of acetyl group production exceeds the capacity of the Krebs cycle, CrAT activity is shifted to the production of acetylcarnitine to prevent depletion of the free CoA pool [151]. Such overproduction is observed during intensive physical exercise in muscle tissue and during ischemia in the heart [121]. The limited availability of free CoA leads to the arrest of the beta-oxidation process and to the accumulation of harmful long-chain acyl–CoA and acylcarnitines in the mitochondria. Therefore, the CrAT mechanism, which ensures a shift towards the production of acetylcarnitine, is considered beneficial.

Shifting CrAT activity towards the production of acetylcarnitine may rescue beta-oxidation by increasing free CoA, but it only partially solves problems of energy deficiency because the acetyl groups do not enter the Krebs cycle for energy production, but rather escape from the mitochondria in the form of acetylcarnitine. The only benefit may be the possibility of pyruvate decarboxylation by the pyruvate dehydrogenase complex (PDC) [96], which produces the equimolar amount of NADH. However, this process may be limited as PDC activity is significantly reduced in the presence of excess acetyl groups. In addition, CrAT activity is shifted towards the production of acetylcarnitine, leading to a depletion of the intramitochondrial free carnitine pool. Carnitine is required for the transport of long-chain acylcarnitines into the mitochondrial matrix, otherwise they accumulate in the mitochondrial intermembrane space and induce detrimental effects on both mitochondrial respiration and pyruvate metabolism. Accordingly, acute carnitine deficiency induced by excessive conversion of free carnitine to acetylcarnitine could lead to detrimental changes in mitochondrial functionality during ischemia and intensive exercise.

#### Acetylcarnitine levels under physiological and pathological conditions

Several studies have shown that long-term use of acetylcarnitine reduces symptoms of chronic fatigue and improves the general condition in patients with chronic fatigue syndrome (Table 1, [79, 81, 142]). Similarly, acetylcarnitine therapy has been shown to improve the general condition in the post-COVID-19 period (Table 1) [115]. However, studies that have measured acetylcarnitine levels in patients with chronic fatigue syndrome or in the post-COVID-19 period have found no differences in blood acetylcarnitine levels compared with healthy subjects [22, 48, 55, 118]. On the other hand, some studies have shown that acetylcarnitine concentrations are significantly increased in patients with heart failure and in non-surviving patients after sepsis (Table 1) [38, 157].

**Table 1:** Blood acetylcarnitine concentrations in healthy and diseased subjects

Conditions	Acetylcarnitine in blood, µmol/L	Comments	References
Healthy	5.5 ± 2.1	Females and males	[105]
	6.2 ± 0.6	Males	[95]
	5.7 ± 0.7	Females	[95]
	6.6 ± 2.3	Females and males	[133]
COVID-19	COVID-19: 7.4 (5.4–11.1)	No difference between groups	[55]
	Non-COVID-19: 6.6 (5.0–9.8)	COVID-19 versus healthy	
COVID-19	Normalized data	No difference between groups	[48]
		healthy versus COVID-19 moderate versus COVID-19 mild	
Chronic fatigue syndrome (CFS)	CFS: 5.5 ± 1.6	No difference between groups	[118]
	Control: 5.8 ± 2.7		
Myalgic encephalomyelitis/chronic fatigue syndrome (ME/CFS)	Male ME/CFS: 4.0 ± 1.8	No difference between groups	[22]
	Male control: 3.9 ± 1.2		
	Female ME/CFS: 4.2 ± 1.9		
	Female control: 3.9 ± 1.2		
Chronic fatigue syndrome (CFS)	Before supplementation	Acetylcarnitine had main effect on mental fatigue	[142]
	5.0 ± 1.6 (non responder)		
	5.0 ± 1.1 (responder)		
	Supplementation with acetylcarnitine	Dosage: 2 g/day, 24 weeks	
	5.50 ± 1.3 (non responder)		
	6.5 ± 1.7 (responder)		
Post-COVID syndrome	Acetylcarnitine was not measured	The combination of exercise and acetylcarnitine is an effective treatment in the management of post-COVID syndrome	[115]
		Dosage: 0.5 g/day, 4 weeks	
Elderly patients with fatigue	Acetylcarnitine was not measured	Acetylcarnitine reduced both physical and mental fatigue in the elderly and improved cognitive status and physical function	[79]
		Dosage: 2 g twice a day for 180 days	
Fatigue in hepatic encephalopathy (HE)	Acetylcarnitine was not measured	Patients with HE treated with acetylcarnitine showed a decrease in the severity of both mental and physical fatigue and an increase in physical activity	[81]
		Dosage: 2 g twice a day for 90 days	
Heart failure (HF)	HF: 10.28 ± 3.5	Acetylcarnitine increased in HF patients	[157]
	Control: 8.05 ± 3.2	Control: patients without HF but with other clinical diseases, with high risk of developing HF	
Sepsis	Survival: 5.08 (3.37–8.8)	Acetylcarnitine increased in patients who did not survive	[38]
	Non-survival: 11.1 (8.2–21.9)		

### Acetylcarnitine supplementation: pharmacodynamics, pharmacokinetics, and clinical efficacy in neuropsychiatric diseases

#### Acetylcarnitine uptake from exogenous sources (e.g., food)

The oral bioavailability of acetylcarnitine, similar to that of carnitine, is relatively low, with some studies reporting absorption rates as low as 5–10% of approximately 2000 mg, which is considered a saturating dose [107, 108]. The relative bioavailability of higher doses has been reported to be even lower [108]. Moreover, acetylcarnitine is rapidly metabolized in the gut by microbiota to trimethylamine, which is further metabolized in the liver to trimethylamine N-oxide, a biomarker of cardiometabolic risk [28]. A considerable amount of research has been dedicated to the various disorders associated with disturbed carnitine and acetylcarnitine homeostasis, but the pharmacokinetics of acetylcarnitine have mostly been studied in healthy subjects and very few studies report carnitine pharmacokinetics in patient groups, most of which are dedicated to studies in hemodialysis patients [108].

**Fig. 3:**
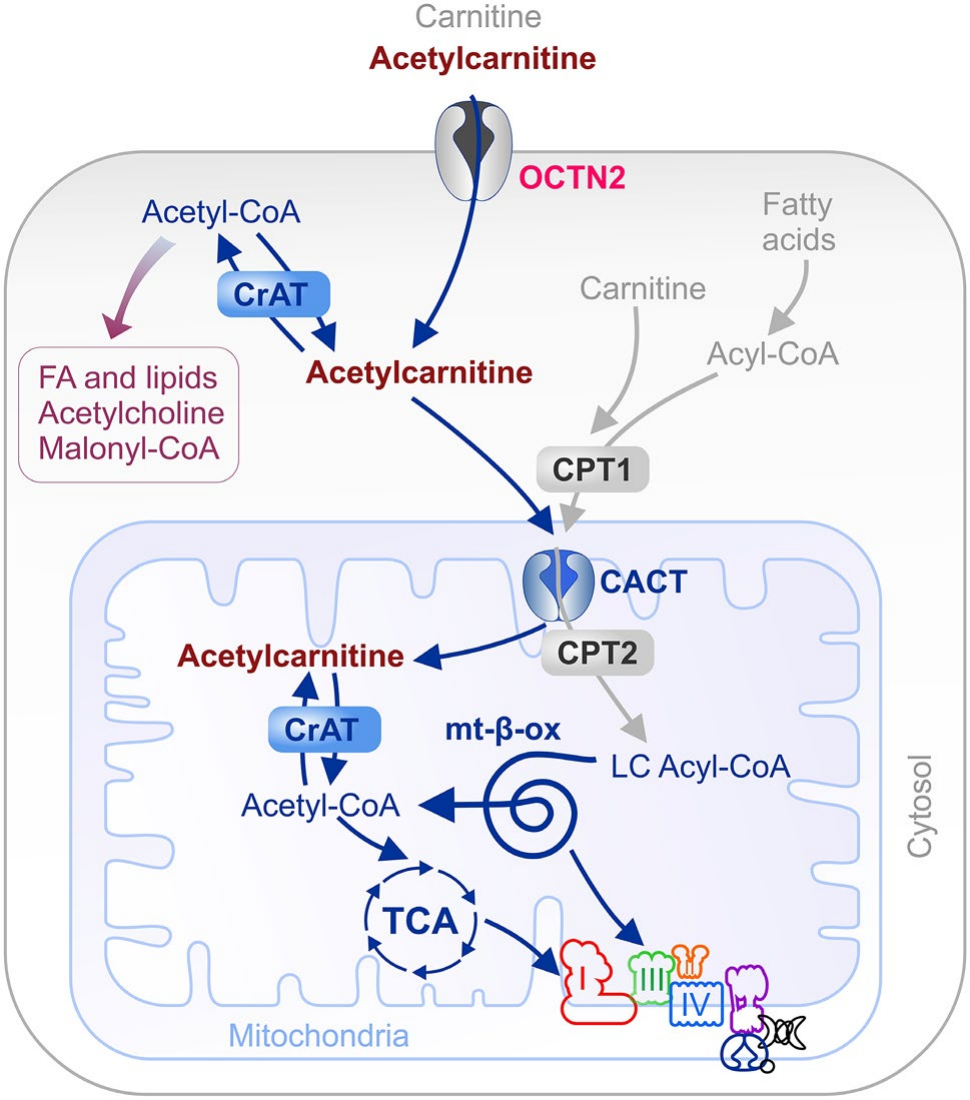
Intracellular effects of acetylcarnitine supplementation

Like other acylcarnitines, acetylcarnitine serves as a transport form for acyl groups, allowing them to cross different membranes with the assistance of various transporters (Fig. 3). OCTN2 (SLC22A5, Organic Cation Transporter Novel 2) is not only known as a main transporter of carnitine but also recognized as an efficient transporter of acetylcarnitine [155]. OCTN2 is localized on various cell membranes and can transport acetylcarnitine into cells as well as ensure acetylcarnitine uptake from the gut and across the blood–brain barrier (BBB) [59]. OCTN2 is differentially expressed in various tissues and plays a major role in regulating carnitine levels within these tissues. OCTN2 is primarily expressed in tissues with high energy demands such as heart, skeletal muscle, liver and kidney. In these tissues, OCTN2 regulates the uptake of carnitine and acetylcarnitine from the blood. OCTN2 is expressed at lower levels in the brain compared to other tissues, therefore the amount of acetylcarnitine transported into the brain would be less active than in other cells [16, 54, 67]. OCTN2 activity can be modulated in response to various physiological and pathological conditions such as exercise, fasting, and metabolic disorders. In addition to OCTN2, organic cation transporter 1 (OCT1, SLC22A1) can also promote the hepatic efflux of acetylcarnitine and contribute to the plasma acetylcarnitine pool [60]. Another transporter, CACT (carnitine-acylcarnitine translocase) is located in the inner mitochondrial membrane and contributes to the exchange of acylcarnitine and longer acylcarnitines for carnitine to ensure acetylcarnitine transfer across the inner mitochondrial membrane [90].

#### Acetylcarnitine metabolism

From a biochemical perspective, acetylcarnitine is an accessible form of the acetyl group that can be taken up from food and transported unchanged to the mitochondrial matrix for energy production via the tricarboxylic acid (TCA) cycle. Prior to metabolism in the mitochondria, the acetyl group is transferred to coenzyme A (CoA) to form acetyl-CoA, which enters the TCA cycle to generate ATP through oxidative phosphorylation. In comparison to other substrates of energy metabolism, acetylcarnitine can be considered as one of the fastest, as it does not require extensive metabolic changes such as glycolysis of fatty acid beta-oxidation, which are complex processes involving many enzymes. Comparing the oxygen required for the oxidation of different substrates to generate ATP, the oxygen efficiency of glucose is generally considered to be more favourable than that of acetyl-CoA, which is more similar to fatty acids. Thus, under conditions of limited oxygen availability, glucose would be a preferred substrate. Overall, acetylcarnitine can improve mitochondrial energy status if supplied to tissues in sufficient quantities.

When acetylcarnitine is injected directly into the brain, most of the acetyl groups were metabolized, as 60% of the injected acetylcarnitine was recovered as exhaled ^14^CO_2_ after 22 h, and the percentage recovered in the brain was 1.9, 1.6, 1.3, and 0.9% at 1, 3, 6, and 22 h, respectively [109]. Some of the acetylcarnitine was incorporated into saturated fatty acids (about 60% of the radioactivity present in the tissues), but it was also found in monounsaturated fatty acids and PUFAs (polyunsaturated fatty acids). The highest level of acetylcarnitine incorporation into brain lipids is observed 1 h after injection and then gradually decreases, suggesting that the metabolic capacity to utilize the acute increase in acetyl groups may be limited and that the excess of acetyl groups is then rapidly incorporated into lipids. In addition, unmetabolized acetyl groups in the brain may be incorporated into various neurotransmitters [114, 150], but it would be an exaggeration to suggest that the availability of acetyl groups has a direct impact on neurotransmitter release at synapses. In the study by Ricciolini and colleagues, labelled [U-^14^C]glucose was not incorporated into PUFAs, unlike [1-^14^C]-acetylcarnitine [109], indicating that the formation of acetyl groups from pyruvate in mitochondria ensures complete metabolism. It is proposed that the beneficial effects of acetylcarnitine include the supply of activated acyl groups for the acylation of membrane phospholipids.

#### Other mechanisms of acetylcarnitine action

Acetylcarnitine has been suggested to protect against oxidative stress through the induction of antioxidant genes and an increase in HO-1 (heme oxygenase 1) expression in vitro and in vivo [17, 18]. However, it is widely accepted that HO-1 is a heat shock protein that is controlled by inflammatory or pro-oxidant states and is induced in various pathologies in response to oxidative stress [1]. In a study in rats, it was demonstrated that aged rats have significantly upregulated protein expression of HO-1 and acetylcarnitine treatment induces further upregulation of HO-1 expression. It has been suggested that the nuclear factor erythroid 2-related factor 2 (Nrf2) pathway is involved in the induction of HO-1 expression by acetylcarnitine [18]. The Nrf2 pathway is typically induced in response to oxidative stress, inflammation, and other cellular stressors [77]. When activated, the Nrf2 pathway upregulates the expression of a wide range of genes that help to protect cells against various stressors such as reactive oxygen species (ROS), electrophiles, and xenobiotics. In addition to its role in cellular defense, the Nrf2 pathway has been implicated in numerous physiological and pathophysiological processes, including aging, neurodegeneration, cancer, and metabolic disorders [77]. Overall, it is not entirely clear what stress-activation mechanisms might be induced by acetylcarnitine to activate Nrf2 pathway and related gene expression. In many in vitro studies, acetylcarnitine concentrations exceed physiological levels by up to 1000-fold [18, 101, 145, 146]. Therefore, acetylcarnitine activities observed in vitro do not always represent pharmacological effects that could be achieved by acetylcarnitine supplementation in humans.

#### Clinical efficacy of acetylcarnitine supplementation in neuropsychiatric diseases

Acetylcarnitine supplementation has been shown to reduce the levels of fatigue-associated with several medical conditions, including aging [79], cancer [84], and chronic hepatitis [80]. There is increasing evidence that acetylcarnitine supplementation may also be effective in neuropsychiatric conditions. For example, acetylcarnitine supplementation improved mental fatigue in an open-label, randomized trial in patients with ME/CFS [142].

Multiple sclerosis (MS) is a neuroinflammatory disease. In addition to neurological symptoms, fatigue is reported by most patients with MS and is associated with a poor quality of life [56]. A randomized cross-over trial in thirty-six MS patients with fatigue showed that 3 months of acetylcarnitine supplementation had a more favourable effect on fatigue severity than amantadine supplementation [131]. However, 1 month of acetylcarnitine supplementation showed only a trend-level improvement in the impact of fatigue on daily life in a placebo-controlled, randomised trial in sixty people with relapsing–remitting MS [70].

MDD is another common neuropsychiatric disease associated with fatigue and cognitive dysfunction. A substantial number of patients do not respond adequately to traditional agents [9], and the non-response rate is estimated to be higher in the immuno-metabolic phenotype [147]. Consistent findings indicate the benefit of acetylcarnitine treatment in general and senile MDD patients [10, 11, 41]. In their meta-analysis combining the results of twelve randomized controlled trials, Veronese and colleagues reported a significant decrease in depression severity after acetylcarnitine supplementation as monotherapy compared with placebo intake or no intervention [143]. The incidence of adverse effects with acetylcarnitine supplementation was comparable to that with placebo and lower than with antidepressants, in line with a previous meta-analysis [85]. Furthermore, the acetylcarnitine supplementation has been reported to be as effective as antidepressants in meta-analyses [64, 143]. To the authors’ knowledge, there are no published results on the effectiveness of acetylcarnitine supplementation in immuno-metabolic depression.

There is preliminary evidence in the literature for the benefit of acetylcarnitine in the treatment of long COVID syndrome. A recent observational case–control study reported improvements in the quality of life, depressive complaints, and pain scores after 1 month of combined physical exercise and acetylcarnitine supplementation compared with physical exercise alone [115]. A recent study of patients with long COVID syndrome reported a decrease in fatigue and an increase in subjective energy levels after 2 weeks of taking a supplement containing 150 mg of acetylcarnitine [93]. These findings suggest that acetylcarnitine supplementation is a promising potential treatment for long COVID syndrome. However, to the authors’ knowledge, there are no ongoing, withdrawn or completed clinical trials investigating the potential benefits of acetylcarnitine supplementation in people with long COVID syndrome. It is imperative that future studies are conducted to assess the effect of acetylcarnitine supplementation on the symptoms associated with long COVID syndrome.

## Conclusion

In conclusion, we have shown in this manuscript, that neuropsychiatric long COVID syndrome shares considerable symptomatic and multi-level biological overlap with other PAIS [23, 62]. Hence, at least a partial overlap in the underlying pathophysiology and thus potential treatment strategies can be assumed for long COVID, PAIS and ME/CFS. In addition to several classical drugs, a large number of dietary interventions and dietary/nutritional supplements, such as amino acids, probiotics, and plant-based extracts, have been tested or proposed for the treatment of long COVID, due to the usually good accessibility, affordability, safety, and easy application of the respective dietary/nutritional supplements [132].

Acetylcarnitine, which, as we have described, plays an important role in mitochondrial energy metabolism, is an FDA-approved dietary supplement that has been studied for the treatment of fatigue in aging, cancer, chronic hepatitis, ME/CFS, MS, and MDD. Treatment with acetylcarnitine alone or as a part of a combined dietary intervention has resulted in reduced levels of fatigue, decreased depression, improved cognitive status, and/or improved physical function in these disorders.

Interestingly, recent pilot studies have reported preliminary evidence of potential benefits of acetylcarnitine supplementation alone or as part of a combined dietary intervention in the treatment of long COVID-associated fatigue, energy metabolism alterations and immune changes [93, 110]. However, there is still no clear evidence if and how acetylcarnitine supplementation alone can improve neuropsychiatric symptoms of long COVID. Given the clinical success of acetylcarnitine supplementation in other fatigue-associated diseases and the small number of currently available treatments for long COVID, we believe that acetylcarnitine may also be a potentially effective dietary supplement for neuropsychiatric long COVID syndrome and call for further action to investigate this hypothesis in further clinical trials.

## Abbreviations

BBB: Blood–brain barrier
CFS: Chronic fatigue syndrome
CNS: Central nervous system
CoA: Coenzyme A
COVID-19: Coronavirus disease 2019
CrAT: Carnitine acetyltransferase
CRP: C-reactive protein
HPA: Hypothalamic–pituitary–adrenal
IDO: Indoleamine 2,3-dioxygenase
IL-2: Interleukin-2
IMD: Immunometabolic depression
MDD: Major depressive disorder
ME: Myalgic encephalomyelitis
mGluR2: Metabotropic glutamate receptor 2
MS: Multiple sclerosis
NMDA: *N*-Methyl-d-aspartate
NMR: Nuclear magnetic resonance
Nrf2: Nuclear factor erythroid 2-related factor 2
OCTN2: Organic cation novel type 2 transporter
OCT1: Organic cation transporter 1
PAIS: Post-acute infection syndromes
PASC: Post-acute sequelae of SARS-CoV-2 infection
PDC: Pyruvate dehydrogenase complex
PGD2S: Prostaglandin D2 synthase gene
QUIN: Quinolinic acid
ROS: Reactive oxygen species
Trp: Tryptophan
TCA: Tricarboxylic acid
HO-1: Heme oxygenase 1
PUFAs: Polyunsaturated fatty acids
CACT: Carnitine-acylcarnitine translocase
MRI: Magnetic resonance imaging
[^18^F]FDG-PET/CT: [^18^F]Fluorodeoxyglucose positron emission tomography/computed tomography
